# Corneal Collagen Cross-Linking for Keratoconus: Systematic Review

**DOI:** 10.1155/2017/8145651

**Published:** 2017-06-11

**Authors:** Hidenaga Kobashi, Shi Song Rong

**Affiliations:** ^1^Department of Ophthalmology, University of Kitasato School of Medicine, Kanagawa, Japan; ^2^Schepens Eye Research Institute, Massachusetts Eye and Ear Infirmary, Department of Ophthalmology, Harvard Medical School, Boston, MA, USA; ^3^Massachusetts Eye and Ear Infirmary, Harvard Medical School, Boston, MA, USA

## Abstract

**Purpose:**

To evaluate the efficacy of collagen cross-linking (CXL) one year after treatment for keratoconus compared to no treatment by summarizing randomized controlled trials (RCTs) using a systematic review.

**Methods:**

Trials meeting the selection criteria were quality appraised, and the data were extracted by two independent authors. The outcome parameters included maximum keratometry (*K*_max_), corneal thickness at the thinnest point, best spectacle-corrected visual acuity (BSCVA), uncorrected visual acuity (UCVA), spherical equivalent (SE) refraction, and cylindrical refraction one year after CXL. We compared the changes in the above parameters with the control group.

**Results:**

We identified five RCTs involving 289 eyes that met the eligibility criteria for this systematic review. The changes in BSCVA from baseline to one year exhibited a significant difference between the two groups. There was no statistically significant difference between the two groups for changes in corneal thickness and cylindrical refraction. We did not conduct a meta-analysis in *K*_max_, UCVA, and SE refraction because their *I*^2^ values were greater than 50%.

**Conclusions:**

According to the systematic review, CXL may be effective in halting the progression of keratoconus for one year under certain conditions, although evidence is limited due to the significant heterogeneity and paucity of RCTs.

## 1. Introduction

Keratoconus is characterized as a bilateral, noninflammatory, progressive corneal ectasia [1]. It results in corneal thinning and protrusion, progressive myopia, and irregular astigmatism. Although only 26.8% of patients with keratoconus progress to requiring corneal transplantation for visual recovery [2], keratoconus remains the most common indication for corneal transplantation surgery [3].

Corneal collagen cross-linking (CXL) was first introduced by Wollensak et al. as a promising technique to slow or stop the progression of keratoconus [4]. In CXL, riboflavin (vitamin B2) is administered in conjunction with ultraviolet A (UVA, 365 nm). The interaction of riboflavin and UVA leads to the formation of reactive oxygen species, which leads to the formation of additional covalent bonds between collagen molecules, with consequent biomechanical stiffening of the cornea [5]. Since the first clinical study was published by Wollensak et al. [4], there have been an increasing number of published studies reporting the safety and efficacy of the treatment in slowing down or halting the progression of keratoconus. CXL received Food and Drug Administration approval in 2016 in the United States [6]. Previous studies, however, are limited by their lack of a control group and relatively short-term follow-up, particularly considering the inherent variability in the course of keratoconus [7] and the limited reproducibility of the measurement of outcome parameters [8]. Several studies focusing on the successful treatment of keratoconus with CXL have been performed and published as randomized controlled trials (RCTs). Previous meta-analyses and a Cochrane review of all published RCTs of CXL for the treatment of keratoconus tried to verify the efficacy of CXL as treatment in stabilizing keratoconus; however, the meta-analysis by Li et al. [9] included one short-term RCT with a follow-up three months postoperatively [10], and the Cochrane review arrived at inconclusive findings due to low quality of evidence and small sample sizes of RCTs conducted until August 2014 [11]. Li et al. [9] calculated the outcomes without adjusting the postoperative time period, and they used two studies from same authors, namely, Wittig-Silva. Sykakis et al. [11] reviewed three RCTs to determine whether there is evidence that CXL is an effective treatment compared to no treatment for halting the progression of keratoconus. However, their Cochrane review was unable to conduct a quantitative synthesis of the evidence because of the small number of RCTs. The two other meta-analyses included comparative and retrospective studies, which may lack evidence [12, 13]. Our hypothesis is that CXL may be effective in halting the progression of keratoconus for long-term follow-ups. The purpose of this study is to evaluate efficacy of CXL one year after treatment of keratoconus compared to no treatment by conducting a systematic review of the literature.

## 2. Materials and Methods

### 2.1. Study Selection

Two reviewers searched the MEDLINE, EMBASE, and the Cochrane Central Register of Controlled Trials (CENTRAL) databases for publications from January 1, 2003, to December 31, 2015. Our search was performed on January 1, 2016. The first published trial report evaluating the effect of CXL in patients with keratoconus was published in 2003 [4]; therefore, we used 2003 as the starting point for the literature search. The keywords in our search strategy include “corneal cross-linking”, “corneal collagen cross-linking”, “collagen cross-linkage”, and “keratoconus”. Two reviewers reviewed the titles and abstracts of the search results and retrieved full-text articles if the title or abstract appeared to meet the eligibility criteria for this review.

### 2.2. Study Inclusion and Exclusion Criteria

Studies were included if they discussed the diagnosis of progressive keratoconus (Amsler-Krumeich grades I and III) [14]. We defined the progression of keratoconus as an increase of at least 0.75 diopter (D) in the steepest keratometry, a degradation of visual acuity, and an increase of 0.75 D or more in the manifest cylinder over the preceding 12 months. We included studies that had a one-year minimum follow-up time and followed the Dresden protocol for CXL. When the same trial was drawn by a screening, we used the most recent trial report. Only studies including human research participants and published in the English language were included. We excluded studies that included patients with a history of corneal surgery and corneal pachymetry less than 300 mm. Articles on corneal collagen cross-linking combined with other treatments, such as topography-guided photorefractive keratectomy or intrastromal corneal ring segments, were excluded. We also excluded cohort studies, case-control studies, and studies that did not use a random method to prospectively assign participants to two groups.

We included trials that compared CXL to contralateral eyes without any treatment or eyes from different keratoconus patients. Eyes that received riboflavin ophthalmic solution alone as the sham control were excluded. All articles that we found were carefully reviewed to select those that reported original clinical data pre- and postoperatively. Data from previously reported cases included in different articles were omitted to avoid duplication of data.

### 2.3. Assessment of Risk of Bias in Included Studies

Two review authors independently assessed the risk of bias of the included studies in accordance with the Cochrane Handbook for Systematic Reviews of Interventions [15] using the following parameters: adequacy of sequence generation; allocation concealment; blinding of participants, personnel, and outcome assessors; incomplete outcome data; and selective outcome reporting. Disagreements were resolved by discussion.

### 2.4. Types of Outcome Measures

Our primary outcomes were the changes in the following parameters between baseline and one-year follow-up:

- Maximum keratometry value (*K*_max_): the steepest keratometry value obtained using topographies of a rotating Scheimpflug camera or computerized videokeratography
- Thinnest corneal thickness: the thickness of the thinnest point using ultrasound pachymetry
- Best spectacle-corrected visual acuity (BSCVA): the visual acuity corrected by only glasses
- Uncorrected visual acuity (UCVA): the visual acuity without correction

Our secondary outcomes were the following:

- Spherical equivalent (SE) refraction: the manifest subjective refraction of the SE
- Cylindrical refraction: the manifest subjective refraction of the cylinder

Best-corrected visual acuity with a contact lens was included in this analysis because the evaluation of visual acuity was limited to BSCVA or UCVA in most previous trials.

### 2.5. Data Extraction

Two reviewers independently extracted data from the included trials using a standardized form. We collected the above outcome measures and details of the interventions, such as setting, sample size, age, mean baseline *K*_max_, control design, and follow-up period. We requested the unpublished data from the corresponding authors of the individual trials via email and waited for their replies for six months.

### 2.6. Assessment of Heterogeneity

We planned to assess heterogeneity by looking at the clinical and methodological diversity of the included studies and by examining the forest plots and* I*^2^ statistics as described in the Cochrane Handbook for Systematic Reviews of Interventions [15].

### 2.7. Data Synthesis and Statistical Analysis

We examined the study characteristics and* I*^2^ statistic as described above. We did not conduct a meta-analysis if there was significant heterogeneity. An* I*^2^ value greater than 50% was considered evidence of significant heterogeneity.

For comparisons where it was appropriate to conduct a meta-analysis, we calculated weighted mean differences and 95% confidence intervals (CIs). We used a fixed-effect model if there were three or fewer studies and a random-effects model if more studies were available. The statistical option used for this analysis was the weighted mean difference for comparing mean changes ± standard deviation values for each parameter from baseline to one-year follow-up between the study and control groups. All statistical analyses were performed with RevMan software (version 5.2, Information Management Systems Group, Cochrane Collaboration).

## 3. Results

### 3.1. Results of the Search

**Figure 1:**
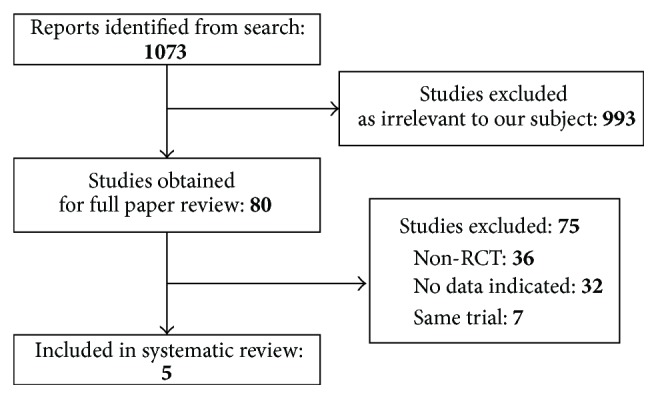
Flow of trial selection. RCT: randomized controlled trial.

There were 1073 articles relevant to the search terms. After screening titles and abstracts, we excluded 993 studies. Of the 80 publications that were initially considered as potentially relevant, we excluded 75 studies because they did not meet the predefined inclusion criteria (Figure 1). Five prospective RCTs involving 289 eyes were included in this systematic review [16–20]. As the same trial, we excluded seven publications which were composed of six studies by Greenstein et al. and one by Wittig-Silva et al. We obtained the primary and secondary outcomes data at one-year follow-up as unpublished information from O'Brart et al. [17] and Lang et al. [19]. Data on our primary and secondary outcomes were available in the papers by Greenstein et al. [16], Wittig-Silva et al. [18], and Seyedian et al. [20]

### 3.2. Characteristics of the Included Studies

Table 1 shows the main characteristics of the included trials. Three [16, 17, 20] of the five trials were studies that used the contralateral eye as the control; the contralateral eye was matched for progression of keratoconus, and age and sex matching were not required. Two studies [18, 19] used two different populations that matched groups for age and progression of keratoconus. No description of the mean age was available in the paper by Greenstein et al. [16]

### 3.3. Primary Outcomes

**Figure 2:**
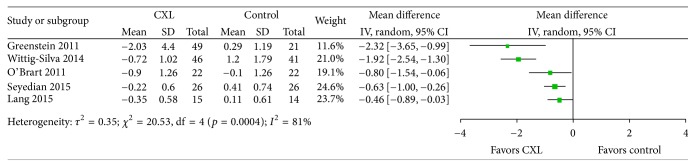
Change in the maximum keratometry value between CXL and control groups. Significant heterogeneity was observed as indicated by an* I*^2^ of 81%. CXL: collagen cross-linking, SD: standard deviation, and CI: confidence interval.

*K*_max_ data were reported by all five included studies (Figure 2). All five studies showed a reduction in *K*_max_ at one year. Two studies were marginally statistically significant while the other three favored CXL. We did not conduct a meta-analysis because* I*^2^ was 81%.

**Figure 3:**
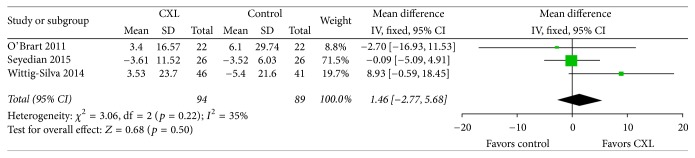
Change in the thinnest corneal thickness between CXL and control groups. No significant heterogeneity was observed as indicated by an* I*^2^ of 35%. There was no significant difference between groups (*p* = 0.50). CXL: collagen cross-linking, SD: standard deviation, and CI: confidence interval.

The thinnest corneal thickness data were reported by three of the five studies that qualified for inclusion in our study (Figure 3). We observed evidence of no significant statistical heterogeneity as indicated by an* I*^2^ of 35%. The thinnest corneal thickness forest plots showed no significant difference in the change after one-year follow-up between the two groups (weighted mean difference = 1.46; 95% CI, −2.27 to 5.68;* p* = 0.50). Wittig-Silva et al. [18] reported that thickness at the thinnest point revealed no significant difference in the eyes after CXL treatment at 12 months, whereas its value decreased in the control eyes. In contrast, O'Brart et al. [17] reported that the thinnest point remained stationary in both post-CXL and control eyes. Thus, whether change in the thinnest point after CXL in keratoconus occurs is controversial.

**Figure 4:**
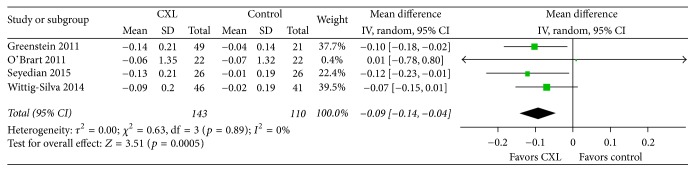
Change in the best spectacle-corrected visual acuity between CXL and control groups. No significant heterogeneity was observed as indicated by an* I*^2^ of 0%. There was a significant difference between groups (*p* = 0.0005). CXL: collagen cross-linking, SD: standard deviation, and CI: confidence interval.

**Figure 5:**
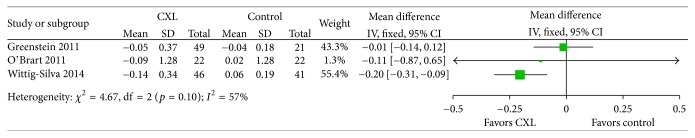
Change in the uncorrected visual acuity between CXL and control groups. Significant heterogeneity was observed as indicated by an* I*^2^ of 57%. CXL: collagen cross-linking, SD: standard deviation, and CI: confidence interval.

BSCVA were reported by four of five studies that qualified for inclusion in our study (Figure 4). We found evidence of no significant statistical heterogeneity as indicated by an* I*^2^ of 0%. The change in BSCVA showed a significant difference between the CXL and control groups based on our data synthesis of four RCTs during the one-year observation period, as the weighted mean difference in the value was −0.09 (CI, −0.14 to −0.04;* p* = 0.0005). The result demonstrated that CXL was favored for BSCVA. However, the value does not seem too clinically meaningful because it is less than a line on an eye chart and is within typical test-retest variability. UCVA were reported by three of five studies that qualified for inclusion in our study (Figure 5). We did not conduct a meta-analysis because* I*^2^ was 57%. Although CXL treatment is not intended to improve visual acuity, the induced changes in corneal topography may result in such improvement secondarily. Among recent studies with 12-month follow-up data, O'Brart et al. [17] reported that BSCVA increased by two lines in six (43%) eyes and by one line in six (20%) eyes. We assume that the results of this analysis of CXL continue to support the efficacy of this treatment in progressive keratoconus, with an improvement in BSCVA 12 months after CXL.

### 3.4. Secondary Outcomes

**Figure 6:**
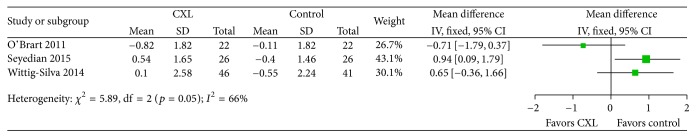
Change in the spherical equivalent refraction between CXL and control groups. Significant heterogeneity was observed as indicated by an* I*^2^ of 66%. CXL: collagen cross-linking, SD: standard deviation, and CI: confidence interval.

**Figure 7:**
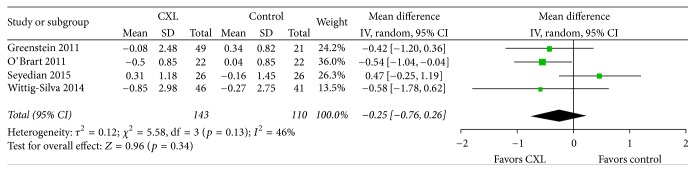
Change in the cylindrical refraction between CXL and control groups. No significant heterogeneity was observed as indicated by an* I*^2^ of 46%. There was no significant difference between groups (*p* = 0.34). CXL: collagen cross-linking, SD: standard deviation, and CI: confidence interval.

The SE refraction data were reported by three of the five studies that qualified for inclusion in our study (Figure 6). We did not conduct a meta-analysis because* I*^2^ was 66%. The cylindrical refraction data were reported by four of the five studies that qualified for inclusion in our study (Figure 7). We observed evidence of no significant statistical heterogeneity as indicated by an* I*^2^ of 46%. The cylindrical refraction forest plots showed no significant difference in the change after one-year follow-up between the two groups (weighted mean difference = −0.25; 95% CI, −0.76 to 0.26;* p* = 0.34). A similar review was recently published [21], which suggested that well-performed long-term RCTs and refinement in techniques were still needed to explore the potential benefit of CXL in slowing or reversing progression of keratoconus.

### 3.5. Quality of the Evidence

The risk of bias in included studies is summarized in Table 2. No disagreements were observed between the two reviewers. Most studies provided insufficient information to determine whether the blind outcome assessment and selective reporting were adequate. In terms of risk of bias, although all five trials were at low risk of bias for sequence generation and allocation concealment, the trials were at high risk of bias for blinding of study participants and personnel. Masking of the investigators collecting the postoperative data was unclear for three out of the five trials. One trial (Wittig-Silva 2014) had a relatively high risk of attrition bias [18].

We assessed heterogeneity by examining* I*^2^ statistic. *K*_max_, UCVA, and SE refraction forest plots had* I*^2^ values greater than 50%, which are considered as statistically significant heterogeneity. We assume that these potential differences between trials may be attributable to the control group in each RCT. Three trials used the other eye of the same patient as the control, but two used different patients. The differences in baseline characteristics of the included patients may also affect the heterogeneity of these studies. We found major differences in the mean baseline *K*_max_ between the included studies.

## 4. Discussion

This systematic review has several limitations that should be taken into account when its results are considered. First, the small number of cases per trial and the total number of cases in this systematic review give these analyses low power. Nevertheless, this review provides more powerful evidence than the individual reports alone, and we are unaware of any other similar systematic reviews. Second, we could only include data from published articles, and bias could be introduced if studies with small or different effects exist but have not been published. Third, in our systematic review, some RCTs used the other eye of the same patient as the control, which might be not appropriate because keratoconus is asymmetrical and there may be differences between an individual's eyes. Jain et al. [22] reviewed the appropriate methodologies of control eyes in the RCT design. However, there were only two RCTs that used different patients as controls. Further RCTs with different patients as controls are required to confirm the present outcomes. Fourth, we did not evaluate the minimum keratometry and intraocular pressure. A previous meta-analysis of RCTs revealed that there was a significant difference in the change in minimum keratometry between CXL and control groups, but not in intraocular pressure [9]. These two parameters might not be critical for the evaluation of CXL as the treatment for keratoconus.

We could calculate the weighted mean difference in some parameters, such as thinnest corneal thickness, BSCVA, and cylindrical refraction because their* I*^2^ values were less than 50%.* I*^2^ is a measurement of heterogeneity. Although 50% could be arbitrary, it was widely used as a cutoff value in judging a higher heterogeneity [23]. A higher heterogeneity could be due to differences in study design, methodology, and true effects. The potential sources of heterogeneity should be further explored for better interpretation of the results from the systematic review.

This systematic review demonstrated that all trials showed a reduction in *K*_max_ during the one-year observation period. The meta-analysis by Li et al. [9] demonstrated that the changes in *K*_max_ and BSCVA were significant in patients undergoing CXL compared with controls. However, their analysis included a three-month follow-up RCT and was limited to trials published in 2014. We updated our review to include an additional two RCTs from 2015. The systematic review and meta-analysis by Chunyu et al. [12] concluded that CXL is effective in halting the progression of keratoconus for at least one year. However, they reviewed studies that included not only RCTs but also prospective controlled studies and retrospective studies citing a lack of evidence. In their review, they used the pre-CXL value for the same eye as the control value. In the present systematic review of RCTs, we included two studies that used different patients as a control, which has sufficient evidence, because keratoconus is a bilateral, asymmetrical disease with varying rates of progression. Sykakis et al. [11] wrote a Cochrane review that assessed the progression at 12 months after CXL in three RCTs published in 2014. By including two RCTs published in 2015, we could represent more updated evidence. The meta-analysis by Meiri et al. [13] in 2016 included population-based prospective and retrospective studies. They did not focus their analysis on RCTs, which could provide the best evidence regarding the efficacy of CXL for a clinical condition. Therefore, based on RCTs, our systematic review could provide a higher level of evidence supporting the use of CXL in the management of keratoconus.

In summary, CXL may be effective in halting the progression of keratoconus for at least one year under certain conditions. However, evidence is limited due to the significant heterogeneity and paucity of RCTs. Further RCTs are necessary to confirm these findings.

## Figures and Tables

**Table 1:** Characteristics of included trials evaluating corneal collagen cross-linking.

Study^*∗*^ (year)	Country	Number of treated eyes	Mean age (years)	Mean baseline *K*_max_ (diopters)	Control design	Follow-up (months)
Greenstein [16] (2011)	United States	49	Not available	60.4	Contralateral eye	12
O'Brart [17] (2011)	United Kingdom	22	29.6	53.9	Contralateral eye	18
Wittig-Silva [18] (2014)	Australia	46	25.7	52.87	Different patients	36
Lang [19] (2015)	Germany	15	29.5	47.3	Different patients	36
Seyedian [20] (2015)	Iran	26	25.6	49.43	Contralateral eye	12

^*∗*^First author.

**Table 2:** Risk of bias in included studies.

Study^*∗*^ (year)	Random sequence generation (selection bias)	Allocation concealment (selection bias)	Blind participants and personnel (performance bias)	Blind outcome assessment (detection bias)	Incomplete outcome data (attrition bias)	Selective reporting (reporting bias)
Greenstein [16] (2011)	Low	Low	High	Unclear	Unclear	Unclear
O'Brart [17] (2011)	Low	Low	High	Low	Low	Unclear
Wittig-Silva [18] (2014)	Low	Low	High	High	High	High
Lang [19] (2015)	Low	Low	High	Unclear	Low	Unclear
Seyedian [20] (2015)	Low	Low	High	Unclear	Low	Unclear

^*∗*^First author.
